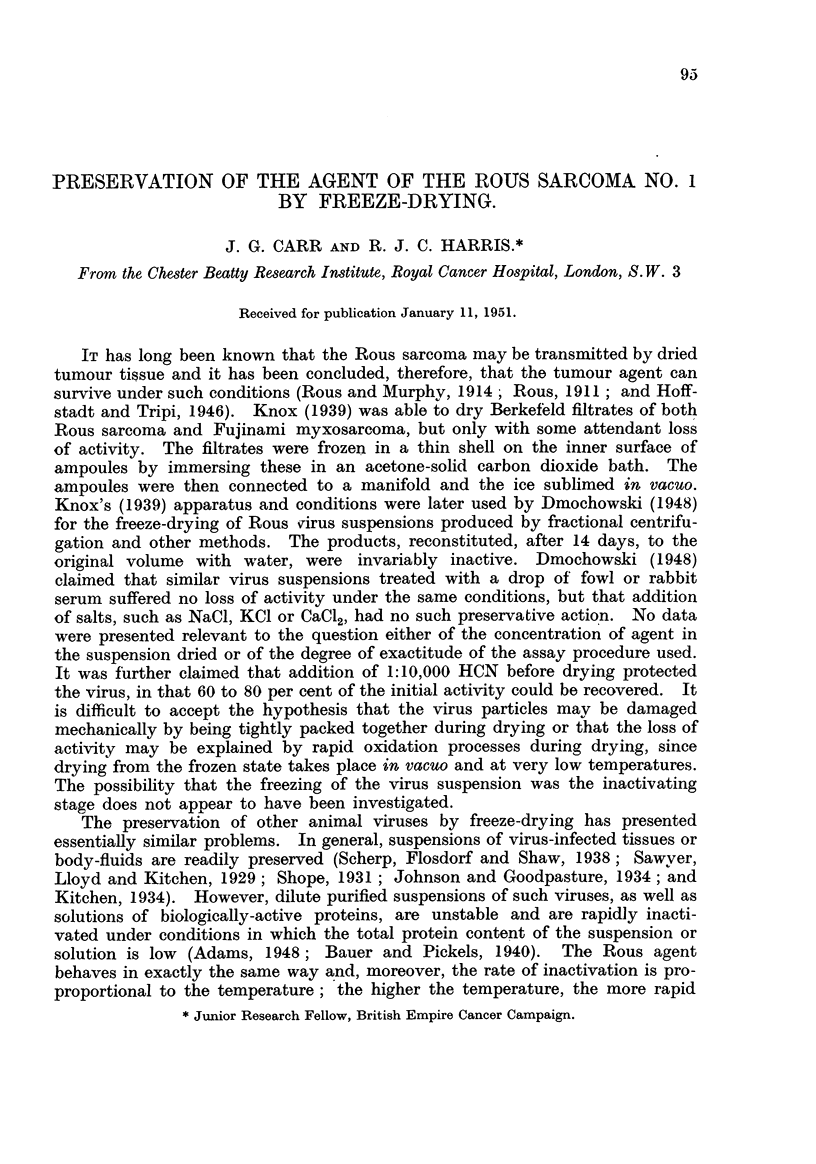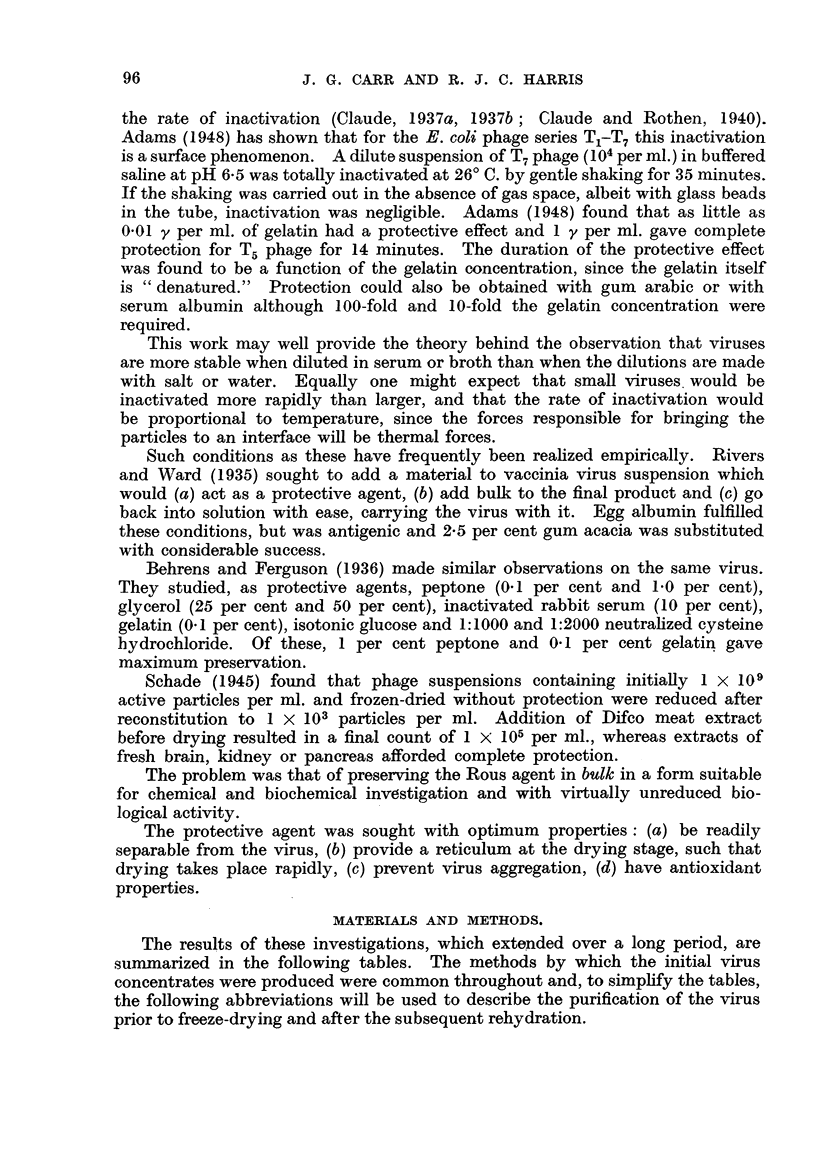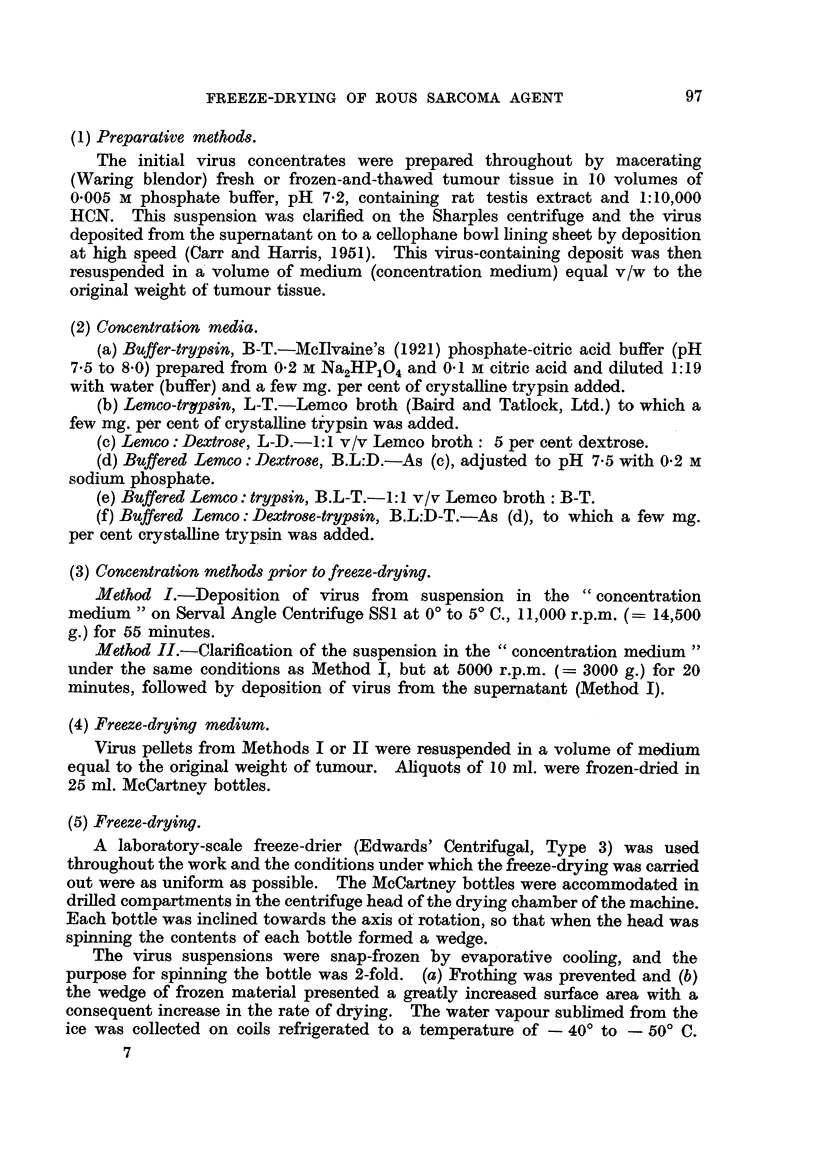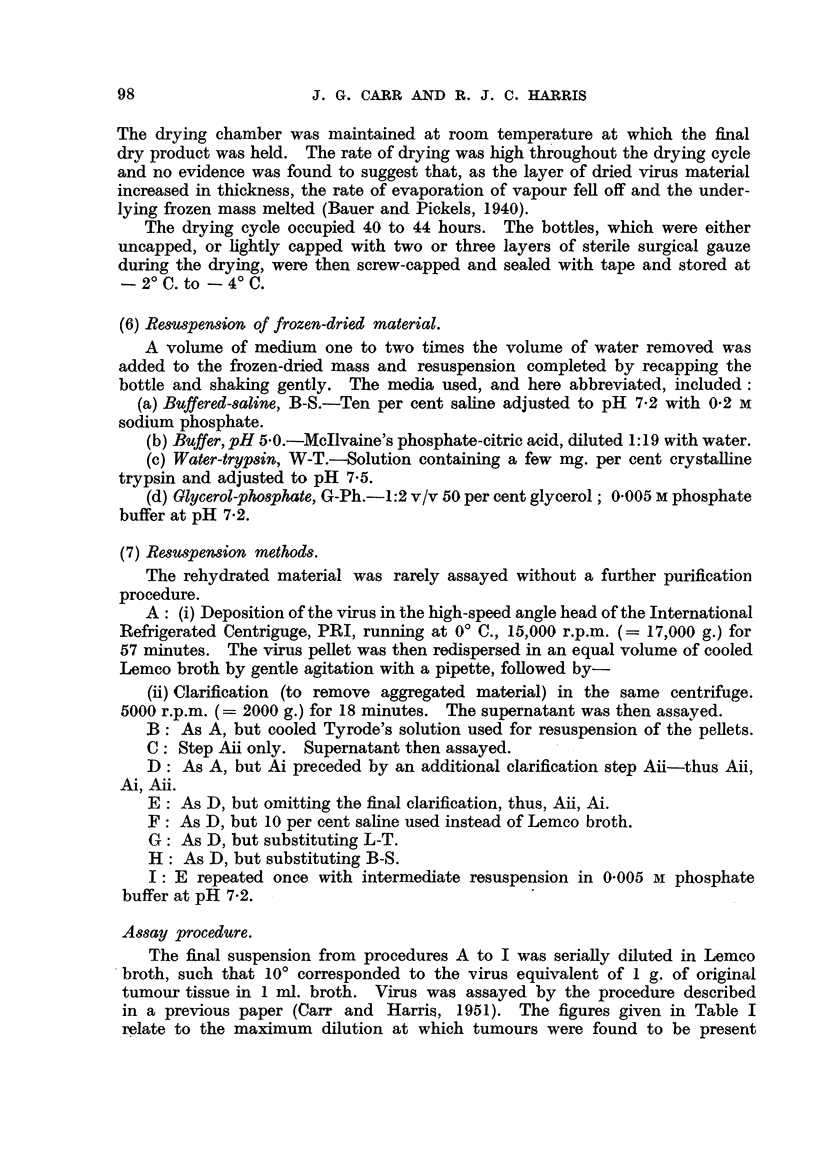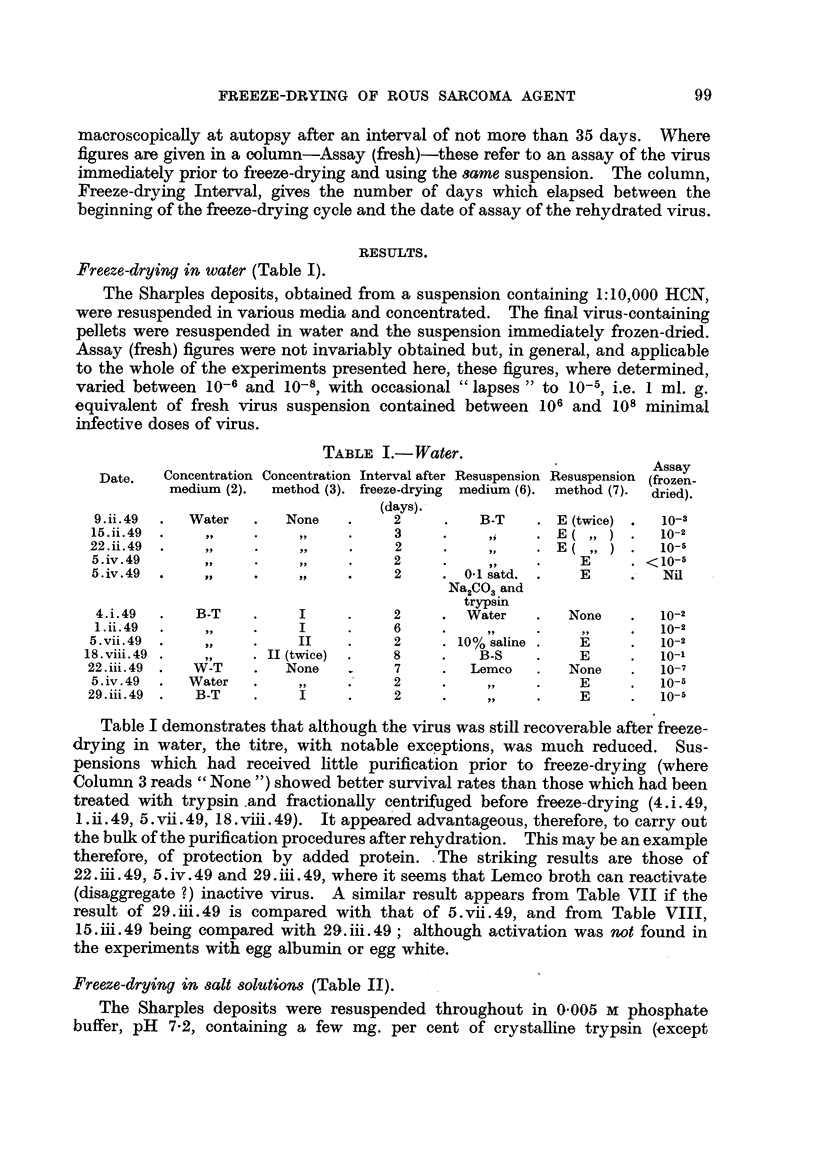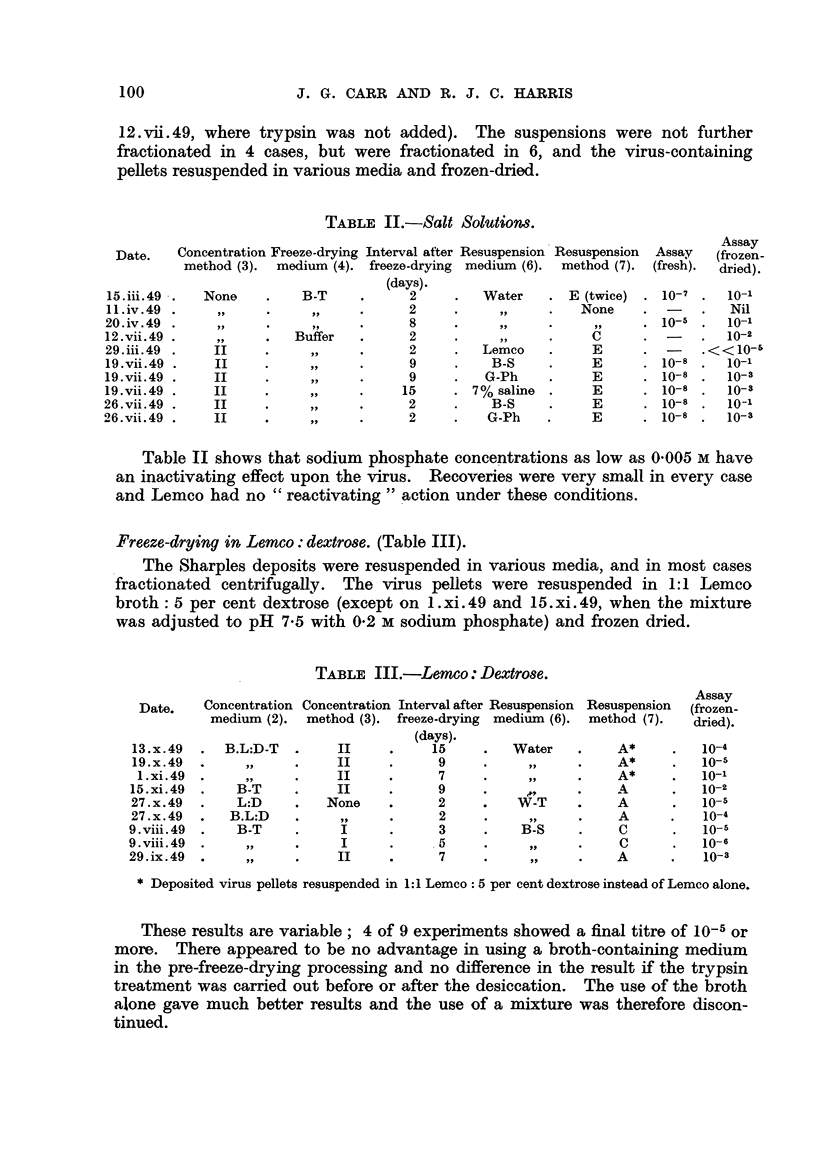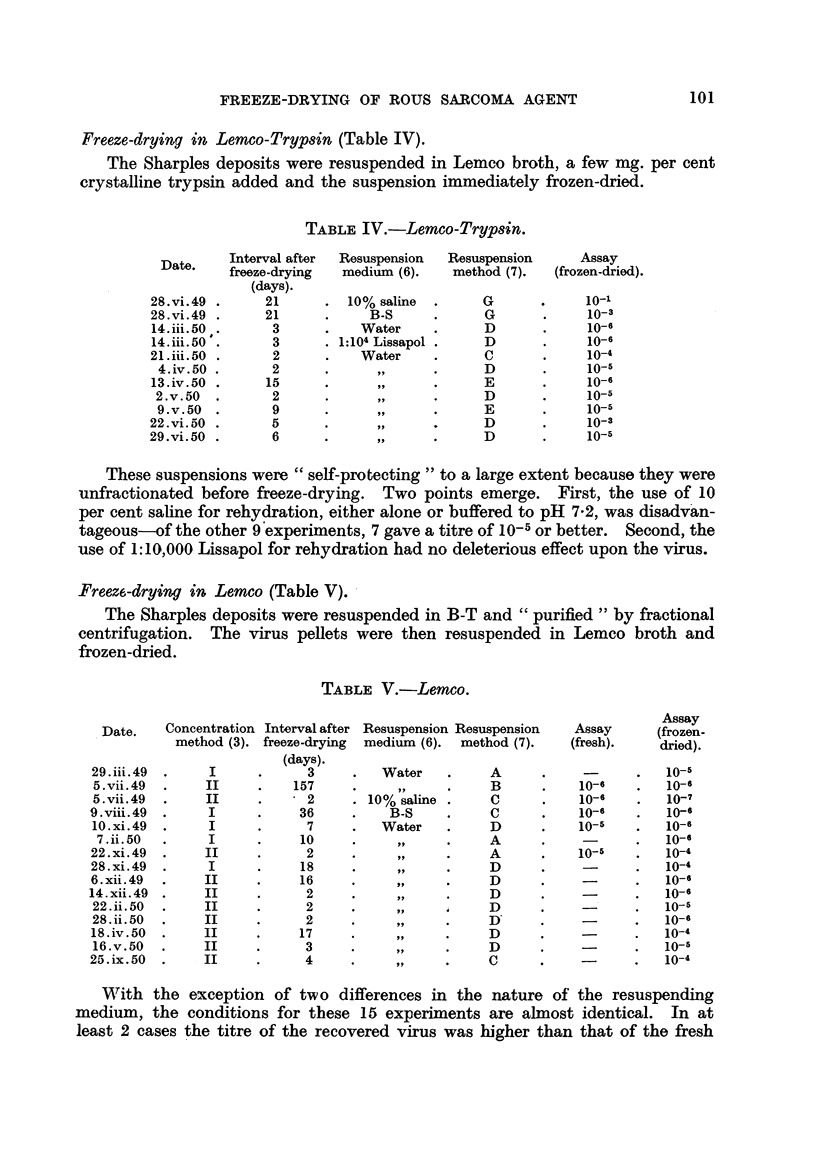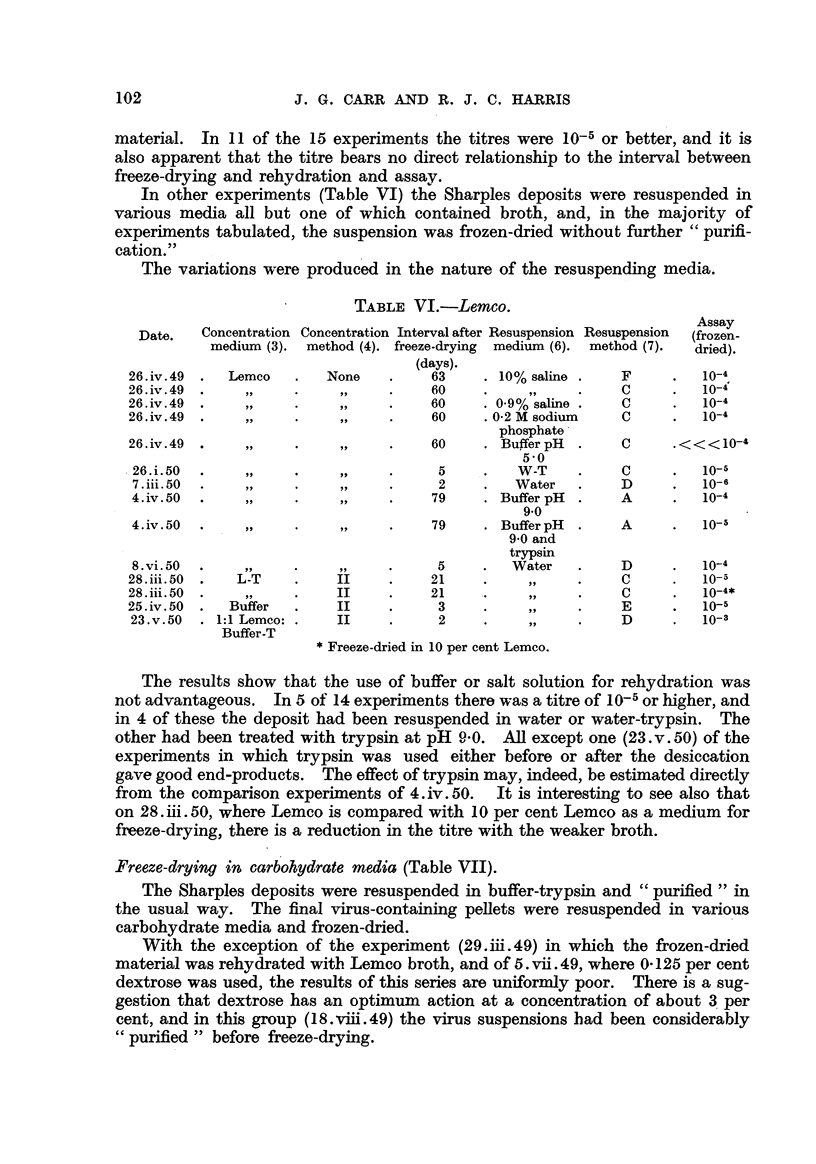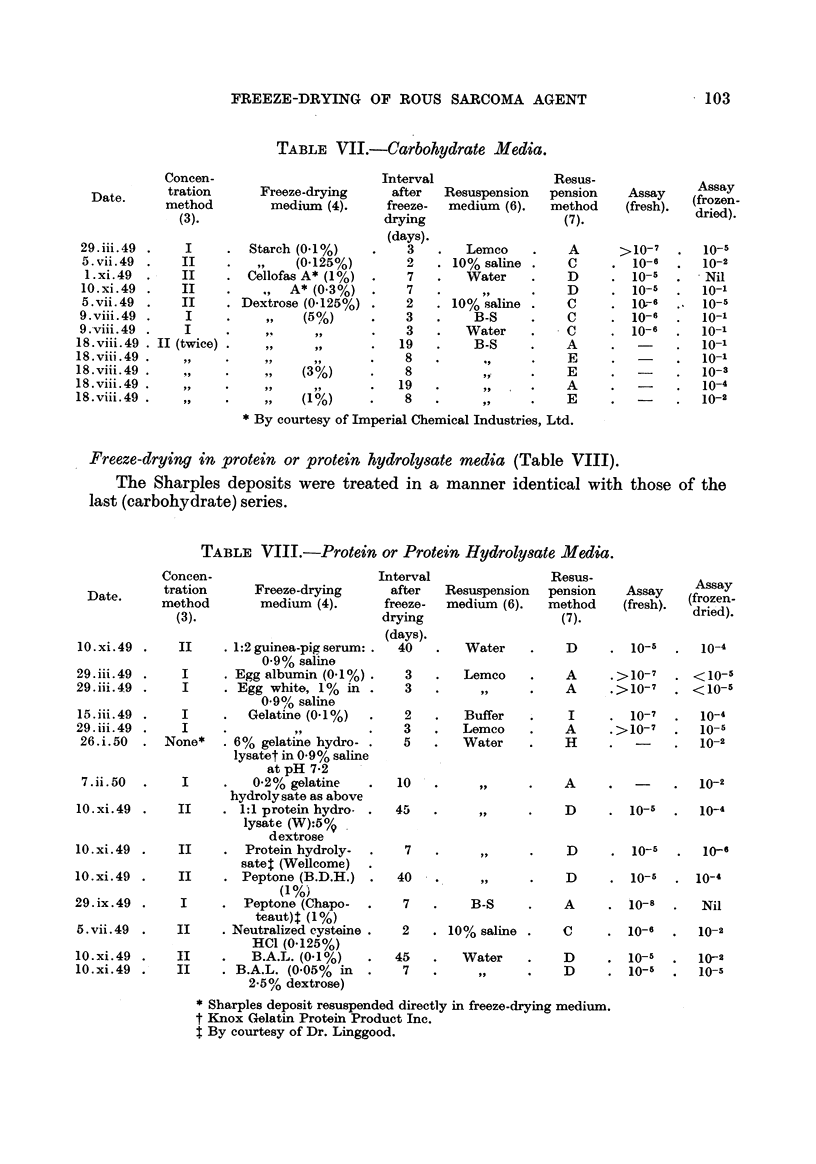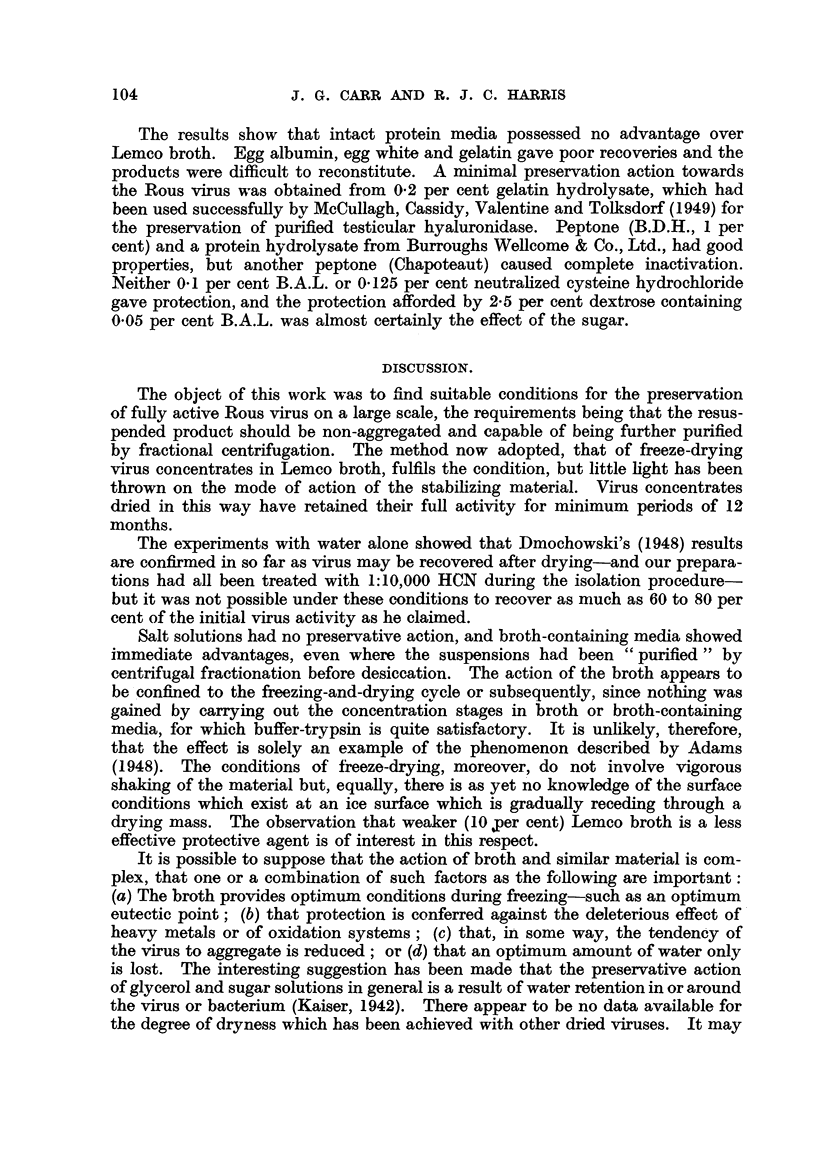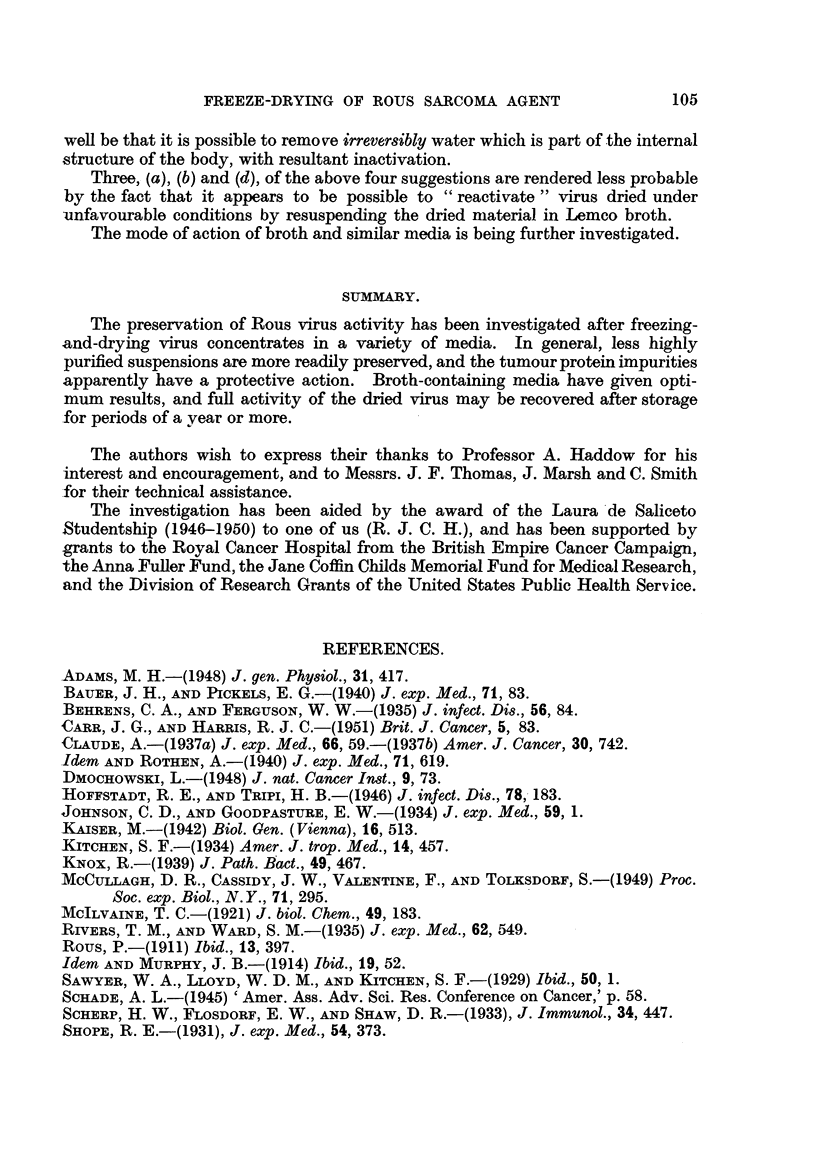# Preservation of the Agent of the Rous Sarcoma No. 1 by Freeze-Drying

**DOI:** 10.1038/bjc.1951.9

**Published:** 1951-03

**Authors:** J. G. Carr, R. J. C. Harris


### PRESERVATION OF THE AGENT OF THE ROUS SARCOMA NO. 1 BY FREEZE-DRYING.

J. G. CARR AND R. J. C. HARRIS.*

*From the Chester Beatty Research Institute, Royal Cancer Hospital, London, S.W. 3*

Received for publication January 11, 1951.

It has long been known that the Rous sarcoma may be transmitted by dried tumour tissue and it has been concluded, therefore, that the tumour agent can survive under such conditions (Rous and Murphy, 1914 ; Rous, 1911 ; and Hoffstadt and Tripi, 1946). Knox (1939) was able to dry Berkefeld filtrates of both Rous sarcoma and Fujinami myxosarcoma, but only with some attendant loss of activity. The filtrates were frozen in a thin shell on the inner surface of ampoules by immersing these in an acetone-solid carbon dioxide bath. The ampoules were then connected to a manifold and the ice sublimed *in vacuo*. Knox's (1939) apparatus and conditions were later used by Dmochowski (1948) for the freeze-drying of Rous virus suspensions produced by fractional centrifugation and other methods. The products, reconstituted, after 14 days, to the original volume with water, were invariably inactive. Dmochowski (1948) claimed that similar virus suspensions treated with a drop of fowl or rabbit serum suffered no loss of activity under the same conditions, but that addition of salts, such as NaCl, KCl or $CaCl_2$, had no such preservative action. No data were presented relevant to the question either of the concentration of agent in the suspension dried or of the degree of exactitude of the assay procedure used. It was further claimed that addition of 1:10,000 HCN before drying protected the virus, in that 60 to 80 per cent of the initial activity could be recovered. It is difficult to accept the hypothesis that the virus particles may be damaged mechanically by being tightly packed together during drying or that the loss of activity may be explained by rapid oxidation processes during drying, since drying from the frozen state takes place *in vacuo* and at very low temperatures. The possibility that the freezing of the virus suspension was the inactivating stage does not appear to have been investigated.

The preservation of other animal viruses by freeze-drying has presented essentially similar problems. In general, suspensions of virus-infected tissues or body-fluids are readily preserved (Scherp, Flosdorf and Shaw, 1938 ; Sawyer, Lloyd and Kitchen, 1929 ; Shope, 1931 ; Johnson and Goodpasture, 1934 ; and Kitchen, 1934). However, dilute purified suspensions of such viruses, as well as solutions of biologically-active proteins, are unstable and are rapidly inactivated under conditions in which the total protein content of the suspension or solution is low (Adams, 1948 ; Bauer and Pickels, 1940). The Rous agent behaves in exactly the same way and, moreover, the rate of inactivation is proproportional to the temperature ; the higher the temperature, the more rapid

* Junior Research Fellow, British Empire Cancer Campaign.

the rate of inactivation (Claude, 1937*a*, 1937*b*; Claude and Rothen, 1940). Adams (1948) has shown that for the *E. coli* phage series $T_1$–$T_7$ this inactivation is a surface phenomenon. A dilute suspension of $T_7$ phage ($10^4$ per ml.) in buffered saline at pH 6·5 was totally inactivated at 26° C. by gentle shaking for 35 minutes. If the shaking was carried out in the absence of gas space, albeit with glass beads in the tube, inactivation was negligible. Adams (1948) found that as little as 0·01 $\gamma$ per ml. of gelatin had a protective effect and 1 $\gamma$ per ml. gave complete protection for $T_5$ phage for 14 minutes. The duration of the protective effect was found to be a function of the gelatin concentration, since the gelatin itself is "denatured." Protection could also be obtained with gum arabic or with serum albumin although 100-fold and 10-fold the gelatin concentration were required.

This work may well provide the theory behind the observation that viruses are more stable when diluted in serum or broth than when the dilutions are made with salt or water. Equally one might expect that small viruses would be inactivated more rapidly than larger, and that the rate of inactivation would be proportional to temperature, since the forces responsible for bringing the particles to an interface will be thermal forces.

Such conditions as these have frequently been realized empirically. Rivers and Ward (1935) sought to add a material to vaccinia virus suspension which would (*a*) act as a protective agent, (*b*) add bulk to the final product and (*c*) go back into solution with ease, carrying the virus with it. Egg albumin fulfilled these conditions, but was antigenic and 2·5 per cent gum acacia was substituted with considerable success.

Behrens and Ferguson (1936) made similar observations on the same virus. They studied, as protective agents, peptone (0·1 per cent and 1·0 per cent), glycerol (25 per cent and 50 per cent), inactivated rabbit serum (10 per cent), gelatin (0·1 per cent), isotonic glucose and 1:1000 and 1:2000 neutralized cysteine hydrochloride. Of these, 1 per cent peptone and 0·1 per cent gelatin gave maximum preservation.

Schade (1945) found that phage suspensions containing initially $1 \times 10^9$ active particles per ml. and frozen-dried without protection were reduced after reconstitution to $1 \times 10^3$ particles per ml. Addition of Difco meat extract before drying resulted in a final count of $1 \times 10^5$ per ml., whereas extracts of fresh brain, kidney or pancreas afforded complete protection.

The problem was that of preserving the Rous agent in *bulk* in a form suitable for chemical and biochemical investigation and with virtually unreduced biological activity.

The protective agent was sought with optimum properties: (*a*) be readily separable from the virus, (*b*) provide a reticulum at the drying stage, such that drying takes place rapidly, (*c*) prevent virus aggregation, (*d*) have antioxidant properties.

### MATERIALS AND METHODS.

The results of these investigations, which extended over a long period, are summarized in the following tables. The methods by which the initial virus concentrates were produced were common throughout and, to simplify the tables, the following abbreviations will be used to describe the purification of the virus prior to freeze-drying and after the subsequent rehydration.

(1) *Preparative methods.*

The initial virus concentrates were prepared throughout by macerating (Waring blendor) fresh or frozen-and-thawed tumour tissue in 10 volumes of 0·005 M phosphate buffer, pH 7·2, containing rat testis extract and 1:10,000 HCN. This suspension was clarified on the Sharples centrifuge and the virus deposited from the supernatant on to a cellophane bowl lining sheet by deposition at high speed (Carr and Harris, 1951). This virus-containing deposit was then resuspended in a volume of medium (concentration medium) equal v/w to the original weight of tumour tissue.

(2) *Concentration media.*

(a) *Buffer-trypsin,* B-T.—McIlvaine's (1921) phosphate-citric acid buffer (pH 7·5 to 8·0) prepared from 0·2 M $Na_2HP_1O_4$ and 0·1 M citric acid and diluted 1:19 with water (buffer) and a few mg. per cent of crystalline trypsin added.

(b) *Lemco-trypsin,* L-T.—Lemco broth (Baird and Tatlock, Ltd.) to which a few mg. per cent of crystalline trypsin was added.

(c) *Lemco : Dextrose,* L-D.—1:1 v/v Lemco broth : 5 per cent dextrose.

(d) *Buffered Lemco : Dextrose,* B.L:D.—As (c), adjusted to pH 7·5 with 0·2 M sodium phosphate.

(e) *Buffered Lemco : trypsin,* B.L-T.—1:1 v/v Lemco broth : B-T.

(f) *Buffered Lemco : Dextrose-trypsin,* B.L:D-T.—As (d), to which a few mg. per cent crystalline trypsin was added.

(3) *Concentration methods prior to freeze-drying.*

*Method I.*—Deposition of virus from suspension in the " concentration medium " on Serval Angle Centrifuge SS1 at 0° to 5° C., 11,000 r.p.m. (= 14,500 g.) for 55 minutes.

*Method II.*—Clarification of the suspension in the " concentration medium " under the same conditions as Method I, but at 5000 r.p.m. (= 3000 g.) for 20 minutes, followed by deposition of virus from the supernatant (Method I).

(4) *Freeze-drying medium.*

Virus pellets from Methods I or II were resuspended in a volume of medium equal to the original weight of tumour. Aliquots of 10 ml. were frozen-dried in 25 ml. McCartney bottles.

(5) *Freeze-drying.*

A laboratory-scale freeze-drier (Edwards' Centrifugal, Type 3) was used throughout the work and the conditions under which the freeze-drying was carried out were as uniform as possible. The McCartney bottles were accommodated in drilled compartments in the centrifuge head of the drying chamber of the machine. Each bottle was inclined towards the axis of rotation, so that when the head was spinning the contents of each bottle formed a wedge.

The virus suspensions were snap-frozen by evaporative cooling, and the purpose for spinning the bottle was 2-fold. (*a*) Frothing was prevented and (*b*) the wedge of frozen material presented a greatly increased surface area with a consequent increase in the rate of drying. The water vapour sublimed from the ice was collected on coils refrigerated to a temperature of — 40° to — 50° C.

The drying chamber was maintained at room temperature at which the final dry product was held. The rate of drying was high throughout the drying cycle and no evidence was found to suggest that, as the layer of dried virus material increased in thickness, the rate of evaporation of vapour fell off and the underlying frozen mass melted (Bauer and Pickels, 1940).

The drying cycle occupied 40 to 44 hours. The bottles, which were either uncapped, or lightly capped with two or three layers of sterile surgical gauze during the drying, were then screw-capped and sealed with tape and stored at − 2° C. to − 4° C.

(6) *Resuspension of frozen-dried material.*

A volume of medium one to two times the volume of water removed was added to the frozen-dried mass and resuspension completed by recapping the bottle and shaking gently. The media used, and here abbreviated, included :

(a) *Buffered-saline,* B-S.—Ten per cent saline adjusted to pH 7·2 with 0·2 M sodium phosphate.

(b) *Buffer, pH* 5·0.—McIlvaine's phosphate-citric acid, diluted 1:19 with water.

(c) *Water-trypsin,* W-T.—Solution containing a few mg. per cent crystalline trypsin and adjusted to pH 7·5.

(d) *Glycerol-phosphate,* G-Ph.—1:2 v/v 50 per cent glycerol ; 0·005 M phosphate buffer at pH 7·2.

(7) *Resuspension methods.*

The rehydrated material was rarely assayed without a further purification procedure.

A : (i) Deposition of the virus in the high-speed angle head of the International Refrigerated Centriguge, PRI, running at 0° C., 15,000 r.p.m. (= 17,000 g.) for 57 minutes. The virus pellet was then redispersed in an equal volume of cooled Lemco broth by gentle agitation with a pipette, followed by—

(ii) Clarification (to remove aggregated material) in the same centrifuge. 5000 r.p.m. (= 2000 g.) for 18 minutes. The supernatant was then assayed.

B : As A, but cooled Tyrode's solution used for resuspension of the pellets.

C : Step Aii only. Supernatant then assayed.

D : As A, but Ai preceded by an additional clarification step Aii—thus Aii, Ai, Aii.

E : As D, but omitting the final clarification, thus, Aii, Ai.

F : As D, but 10 per cent saline used instead of Lemco broth.

G : As D, but substituting L-T.

H : As D, but substituting B-S.

I : E repeated once with intermediate resuspension in 0·005 M phosphate buffer at pH 7·2.

*Assay procedure.*

The final suspension from procedures A to I was serially diluted in Lemco broth, such that 10° corresponded to the virus equivalent of 1 g. of original tumour tissue in 1 ml. broth. Virus was assayed by the procedure described in a previous paper (Carr and Harris, 1951). The figures given in Table I relate to the maximum dilution at which tumours were found to be present macroscopically at autopsy after an interval of not more than 35 days. Where figures are given in a column—Assay (fresh)—these refer to an assay of the virus immediately prior to freeze-drying and using the *same* suspension. The column, Freeze-drying Interval, gives the number of days which elapsed between the beginning of the freeze-drying cycle and the date of assay of the rehydrated virus.

### RESULTS.

*Freeze-drying in water* (Table I).

The Sharples deposits, obtained from a suspension containing 1:10,000 HCN, were resuspended in various media and concentrated. The final virus-containing pellets were resuspended in water and the suspension immediately frozen-dried. Assay (fresh) figures were not invariably obtained but, in general, and applicable to the whole of the experiments presented here, these figures, where determined, varied between $10^{-6}$ and $10^{-8}$, with occasional "lapses" to $10^{-5}$, i.e. 1 ml. g. equivalent of fresh virus suspension contained between $10^{6}$ and $10^{8}$ minimal infective doses of virus.

TABLE I.—*Water.*

| Date. | Concentration medium (2). | Concentration method (3). | Interval after freeze-drying (days). | Resuspension medium (6). | Resuspension method (7). | Assay (frozen-dried). |
|---|---|---|---|---|---|---|
| 9.ii.49 | Water | None | 2 | B-T | E (twice) | $10^{-3}$ |
| 15.ii.49 | ,, | ,, | 3 | ,, | E ( ,, ) | $10^{-2}$ |
| 22.ii.49 | ,, | ,, | 2 | ,, | E ( ,, ) | $10^{-5}$ |
| 5.iv.49 | ,, | ,, | 2 | ,, | E | $<10^{-5}$ |
| 5.iv.49 | ,, | ,, | 2 | 0·1 satd. $Na_2CO_3$ and trypsin | E | Nil |
| 4.i.49 | B-T | I | 2 | Water | None | $10^{-2}$ |
| 1.ii.49 | ,, | I | 6 | ,, | ,, | $10^{-2}$ |
| 5.vii.49 | ,, | II | 2 | 10% saline | E | $10^{-2}$ |
| 18.viii.49 | ,, | II (twice) | 8 | B-S | E | $10^{-1}$ |
| 22.iii.49 | W-T | None | 7 | Lemco | None | $10^{-7}$ |
| 5.iv.49 | Water | ,, | 2 | ,, | E | $10^{-5}$ |
| 29.iii.49 | B-T | I | 2 | ,, | E | $10^{-5}$ |

Table I demonstrates that although the virus was still recoverable after freeze-drying in water, the titre, with notable exceptions, was much reduced. Suspensions which had received little purification prior to freeze-drying (where Column 3 reads "None") showed better survival rates than those which had been treated with trypsin and fractionally centrifuged before freeze-drying (4.i.49, 1.ii.49, 5.vii.49, 18.viii.49). It appeared advantageous, therefore, to carry out the bulk of the purification procedures after rehydration. This may be an example therefore, of protection by added protein. The striking results are those of 22.iii.49, 5.iv.49 and 29.iii.49, where it seems that Lemco broth can reactivate (disaggregate ?) inactive virus. A similar result appears from Table VII if the result of 29.iii.49 is compared with that of 5.vii.49, and from Table VIII, 15.iii.49 being compared with 29.iii.49 ; although activation was *not* found in the experiments with egg albumin or egg white.

*Freeze-drying in salt solutions* (Table II).

The Sharples deposits were resuspended throughout in 0·005 M phosphate buffer, pH 7·2, containing a few mg. per cent of crystalline trypsin (except

12.vii.49, where trypsin was not added). The suspensions were not further fractionated in 4 cases, but were fractionated in 6, and the virus-containing pellets resuspended in various media and frozen-dried.

TABLE II.—*Salt Solutions.*

| Date. | Concentration method (3). | Freeze-drying medium (4). | Interval after freeze-drying (days). | Resuspension medium (6). | Resuspension method (7). | Assay (fresh). | Assay (frozen-dried). |
|---|---|---|---|---|---|---|---|
| 15.iii.49 | None | B-T | 2 | Water | E (twice) | $10^{-7}$ | $10^{-1}$ |
| 11.iv.49 | ” | ” | 2 | ” | None | — | Nil |
| 20.iv.49 | ” | ” | 8 | ” | ” | $10^{-5}$ | $10^{-1}$ |
| 12.vii.49 | ” | Buffer | 2 | ” | C | — | $10^{-2}$ |
| 29.iii.49 | II | ” | 2 | Lemco | E | — | $<<10^{-5}$ |
| 19.vii.49 | II | ” | 9 | B-S | E | $10^{-8}$ | $10^{-1}$ |
| 19.vii.49 | II | ” | 9 | G-Ph | E | $10^{-8}$ | $10^{-3}$ |
| 19.vii.49 | II | ” | 15 | 7% saline | E | $10^{-8}$ | $10^{-3}$ |
| 26.vii.49 | II | ” | 2 | B-S | E | $10^{-8}$ | $10^{-1}$ |
| 26.vii.49 | II | ” | 2 | G-Ph | E | $10^{-8}$ | $10^{-3}$ |

Table II shows that sodium phosphate concentrations as low as 0·005 M have an inactivating effect upon the virus. Recoveries were very small in every case and Lemco had no “ reactivating ” action under these conditions.

*Freeze-drying in Lemco : dextrose.* (Table III).

The Sharples deposits were resuspended in various media, and in most cases fractionated centrifugally. The virus pellets were resuspended in 1:1 Lemco broth : 5 per cent dextrose (except on 1.xi.49 and 15.xi.49, when the mixture was adjusted to pH 7·5 with 0·2 M sodium phosphate) and frozen dried.

TABLE III.—*Lemco : Dextrose.*

| Date. | Concentration medium (2). | Concentration method (3). | Interval after freeze-drying (days). | Resuspension medium (6). | Resuspension method (7). | Assay (frozen-dried). |
|---|---|---|---|---|---|---|
| 13.x.49 | B.L:D-T | II | 15 | Water | A* | $10^{-4}$ |
| 19.x.49 | ” | II | 9 | ” | A* | $10^{-5}$ |
| 1.xi.49 | ” | II | 7 | ” | A* | $10^{-1}$ |
| 15.xi.49 | B-T | II | 9 | ” | A | $10^{-2}$ |
| 27.x.49 | L:D | None | 2 | W-T | A | $10^{-5}$ |
| 27.x.49 | B.L:D | ” | 2 | ” | A | $10^{-4}$ |
| 9.viii.49 | B-T | I | 3 | B-S | C | $10^{-5}$ |
| 9.viii.49 | ” | I | 5 | ” | C | $10^{-6}$ |
| 29.ix.49 | ” | II | 7 | ” | A | $10^{-3}$ |

* Deposited virus pellets resuspended in 1:1 Lemco : 5 per cent dextrose instead of Lemco alone.

These results are variable ; 4 of 9 experiments showed a final titre of $10^{-5}$ or more. There appeared to be no advantage in using a broth-containing medium in the pre-freeze-drying processing and no difference in the result if the trypsin treatment was carried out before or after the desiccation. The use of the broth alone gave much better results and the use of a mixture was therefore discontinued.

*Freeze-drying in Lemco-Trypsin* (Table IV).

The Sharples deposits were resuspended in Lemco broth, a few mg. per cent crystalline trypsin added and the suspension immediately frozen-dried.

TABLE IV.—*Lemco-Trypsin.*

| Date. | Interval after freeze-drying (days). | Resuspension medium (6). | Resuspension method (7). | Assay (frozen-dried). |
|---|---|---|---|---|
| 28.vi.49 | 21 | 10% saline | G | $10^{-1}$ |
| 28.vi.49 | 21 | B-S | G | $10^{-3}$ |
| 14.iii.50 | 3 | Water | D | $10^{-6}$ |
| 14.iii.50 | 3 | 1:10⁴ Lissapol | D | $10^{-6}$ |
| 21.iii.50 | 2 | Water | C | $10^{-4}$ |
| 4.iv.50 | 2 | „ | D | $10^{-5}$ |
| 13.iv.50 | 15 | „ | E | $10^{-6}$ |
| 2.v.50 | 2 | „ | D | $10^{-5}$ |
| 9.v.50 | 9 | „ | E | $10^{-5}$ |
| 22.vi.50 | 5 | „ | D | $10^{-3}$ |
| 29.vi.50 | 6 | „ | D | $10^{-5}$ |

These suspensions were " self-protecting " to a large extent because they were unfractionated before freeze-drying. Two points emerge. First, the use of 10 per cent saline for rehydration, either alone or buffered to pH 7·2, was disadvantageous—of the other 9 experiments, 7 gave a titre of $10^{-5}$ or better. Second, the use of 1:10,000 Lissapol for rehydration had no deleterious effect upon the virus.

*Freeze-drying in Lemco* (Table V).

The Sharples deposits were resuspended in B-T and " purified " by fractional centrifugation. The virus pellets were then resuspended in Lemco broth and frozen-dried.

TABLE V.—*Lemco.*

| Date. | Concentration method (3). | Interval after freeze-drying (days). | Resuspension medium (6). | Resuspension method (7). | Assay (fresh). | Assay (frozen-dried). |
|---|---|---|---|---|---|---|
| 29.iii.49 | I | 3 | Water | A | — | $10^{-5}$ |
| 5.vii.49 | II | 157 | „ | B | $10^{-6}$ | $10^{-6}$ |
| 5.vii.49 | II | 2 | 10% saline | C | $10^{-6}$ | $10^{-7}$ |
| 9.viii.49 | I | 36 | B-S | C | $10^{-6}$ | $10^{-6}$ |
| 10.xi.49 | I | 7 | Water | D | $10^{-5}$ | $10^{-6}$ |
| 7.ii.50 | I | 10 | „ | A | — | $10^{-6}$ |
| 22.xi.49 | II | 2 | „ | A | $10^{-5}$ | $10^{-4}$ |
| 28.xi.49 | I | 18 | „ | D | — | $10^{-4}$ |
| 6.xii.49 | II | 16 | „ | D | — | $10^{-6}$ |
| 14.xii.49 | II | 2 | „ | D | — | $10^{-6}$ |
| 22.ii.50 | II | 2 | „ | D | — | $10^{-5}$ |
| 28.ii.50 | II | 2 | „ | D | — | $10^{-6}$ |
| 18.iv.50 | II | 17 | „ | D | — | $10^{-4}$ |
| 16.v.50 | II | 3 | „ | D | — | $10^{-5}$ |
| 25.ix.50 | II | 4 | „ | C | — | $10^{-4}$ |

With the exception of two differences in the nature of the resuspending medium, the conditions for these 15 experiments are almost identical. In at least 2 cases the titre of the recovered virus was higher than that of the fresh material. In 11 of the 15 experiments the titres were $10^{-5}$ or better, and it is also apparent that the titre bears no direct relationship to the interval between freeze-drying and rehydration and assay.

In other experiments (Table VI) the Sharples deposits were resuspended in various media all but one of which contained broth, and, in the majority of experiments tabulated, the suspension was frozen-dried without further " purification."

The variations were produced in the nature of the resuspending media.

TABLE VI.—*Lemco.*

| Date. | Concentration medium (3). | Concentration method (4). | Interval after freeze-drying (days). | Resuspension medium (6). | Resuspension method (7). | Assay (frozen-dried). |
|---|---|---|---|---|---|---|
| 26.iv.49 | Lemco | None | 63 | 10% saline | F | $10^{-4}$ |
| 26.iv.49 | ,, | ,, | 60 | ,, | C | $10^{-4}$ |
| 26.iv.49 | ,, | ,, | 60 | 0·9% saline | C | $10^{-4}$ |
| 26.iv.49 | ,, | ,, | 60 | 0·2 M sodium phosphate | C | $10^{-4}$ |
| 26.iv.49 | ,, | ,, | 60 | Buffer pH 5·0 | C | $<<<10^{-4}$ |
| 26.i.50 | ,, | ,, | 5 | W-T | C | $10^{-5}$ |
| 7.iii.50 | ,, | ,, | 2 | Water | D | $10^{-6}$ |
| 4.iv.50 | ,, | ,, | 79 | Buffer pH 9·0 | A | $10^{-4}$ |
| 4.iv.50 | ,, | ,, | 79 | Buffer pH 9·0 and trypsin | A | $10^{-5}$ |
| 8.vi.50 | ,, | ,, | 5 | Water | D | $10^{-4}$ |
| 28.iii.50 | L-T | II | 21 | ,, | C | $10^{-5}$ |
| 28.iii.50 | ,, | II | 21 | ,, | C | $10^{-4}$* |
| 25.iv.50 | Buffer | II | 3 | ,, | E | $10^{-5}$ |
| 23.v.50 | 1:1 Lemco: Buffer-T | II | 2 | ,, | D | $10^{-3}$ |

* Freeze-dried in 10 per cent Lemco.

The results show that the use of buffer or salt solution for rehydration was not advantageous. In 5 of 14 experiments there was a titre of $10^{-5}$ or higher, and in 4 of these the deposit had been resuspended in water or water-trypsin. The other had been treated with trypsin at pH 9·0. All except one (23.v.50) of the experiments in which trypsin was used either before or after the desiccation gave good end-products. The effect of trypsin may, indeed, be estimated directly from the comparison experiments of 4.iv.50. It is interesting to see also that on 28.iii.50, where Lemco is compared with 10 per cent Lemco as a medium for freeze-drying, there is a reduction in the titre with the weaker broth.

*Freeze-drying in carbohydrate media* (Table VII).

The Sharples deposits were resuspended in buffer-trypsin and " purified " in the usual way. The final virus-containing pellets were resuspended in various carbohydrate media and frozen-dried.

With the exception of the experiment (29.iii.49) in which the frozen-dried material was rehydrated with Lemco broth, and of 5.vii.49, where 0·125 per cent dextrose was used, the results of this series are uniformly poor. There is a suggestion that dextrose has an optimum action at a concentration of about 3 per cent, and in this group (18.viii.49) the virus suspensions had been considerably " purified " before freeze-drying.

TABLE VII.—*Carbohydrate Media.*

| Date. | Concentration method (3). | Freeze-drying medium (4). | Interval after freeze-drying (days). | Resuspension medium (6). | Resuspension method (7). | Assay (fresh). | Assay (frozen-dried). |
|---|---|---|---|---|---|---|---|
| 29.iii.49 | I | Starch (0·1%) | 3 | Lemco | A | $>10^{-7}$ | $10^{-5}$ |
| 5.vii.49 | II | ,, (0·125%) | 2 | 10% saline | C | $10^{-6}$ | $10^{-2}$ |
| 1.xi.49 | II | Cellofas A* (1%) | 7 | Water | D | $10^{-5}$ | Nil |
| 10.xi.49 | II | ,, A* (0·3%) | 7 | ,, | D | $10^{-5}$ | $10^{-1}$ |
| 5.vii.49 | II | Dextrose (0·125%) | 2 | 10% saline | C | $10^{-6}$ | $10^{-5}$ |
| 9.viii.49 | I | ,, (5%) | 3 | B-S | C | $10^{-6}$ | $10^{-1}$ |
| 9.viii.49 | I | ,, ,, | 3 | Water | C | $10^{-6}$ | $10^{-1}$ |
| 18.viii.49 | II (twice) | ,, ,, | 19 | B-S | A | — | $10^{-1}$ |
| 18.viii.49 | ,, | ,, ,, | 8 | ,, | E | — | $10^{-1}$ |
| 18.viii.49 | ,, | ,, (3%) | 8 | ,, | E | — | $10^{-3}$ |
| 18.viii.49 | ,, | ,, ,, | 19 | ,, | A | — | $10^{-4}$ |
| 18.viii.49 | ,, | ,, (1%) | 8 | ,, | E | — | $10^{-2}$ |

* By courtesy of Imperial Chemical Industries, Ltd.

*Freeze-drying in protein or protein hydrolysate media* (Table VIII).

The Sharples deposits were treated in a manner identical with those of the last (carbohydrate) series.

TABLE VIII.—*Protein or Protein Hydrolysate Media.*

| Date. | Concentration method (3). | Freeze-drying medium (4). | Interval after freeze-drying (days). | Resuspension medium (6). | Resuspension method (7). | Assay (fresh). | Assay (frozen-dried). |
|---|---|---|---|---|---|---|---|
| 10.xi.49 | II | 1:2 guinea-pig serum: 0·9% saline | 40 | Water | D | $10^{-5}$ | $10^{-4}$ |
| 29.iii.49 | I | Egg albumin (0·1%) | 3 | Lemco | A | $>10^{-7}$ | $<10^{-5}$ |
| 29.iii.49 | I | Egg white, 1% in 0·9% saline | 3 | ,, | A | $>10^{-7}$ | $<10^{-5}$ |
| 15.iii.49 | I | Gelatine (0·1%) | 2 | Buffer | I | $10^{-7}$ | $10^{-4}$ |
| 29.iii.49 | I | ,, | 3 | Lemco | A | $>10^{-7}$ | $10^{-5}$ |
| 26.i.50 | None* | 6% gelatine hydrolysate† in 0·9% saline at pH 7·2 | 5 | Water | H | — | $10^{-2}$ |
| 7.ii.50 | I | 0·2% gelatine hydrolysate as above | 10 | ,, | A | — | $10^{-2}$ |
| 10.xi.49 | II | 1:1 protein hydrolysate (W):5% dextrose | 45 | ,, | D | $10^{-5}$ | $10^{-4}$ |
| 10.xi.49 | II | Protein hydrolysate‡ (Wellcome) | 7 | ,, | D | $10^{-5}$ | $10^{-6}$ |
| 10.xi.49 | II | Peptone (B.D.H.) (1%) | 40 | ,, | D | $10^{-5}$ | $10^{-4}$ |
| 29.ix.49 | I | Peptone (Chapoteaut)‡ (1%) | 7 | B-S | A | $10^{-8}$ | Nil |
| 5.vii.49 | II | Neutralized cysteine HCl (0·125%) | 2 | 10% saline | C | $10^{-6}$ | $10^{-2}$ |
| 10.xi.49 | II | B.A.L. (0·1%) | 45 | Water | D | $10^{-5}$ | $10^{-2}$ |
| 10.xi.49 | II | B.A.L. (0·05% in 2·5% dextrose) | 7 | ,, | D | $10^{-5}$ | $10^{-5}$ |

* Sharples deposit resuspended directly in freeze-drying medium.
† Knox Gelatin Protein Product Inc.
‡ By courtesy of Dr. Linggood.

The results show that intact protein media possessed no advantage over Lemco broth. Egg albumin, egg white and gelatin gave poor recoveries and the products were difficult to reconstitute. A minimal preservation action towards the Rous virus was obtained from 0·2 per cent gelatin hydrolysate, which had been used successfully by McCullagh, Cassidy, Valentine and Tolksdorf (1949) for the preservation of purified testicular hyaluronidase. Peptone (B.D.H., 1 per cent) and a protein hydrolysate from Burroughs Wellcome & Co., Ltd., had good properties, but another peptone (Chapoteaut) caused complete inactivation. Neither 0·1 per cent B.A.L. or 0·125 per cent neutralized cysteine hydrochloride gave protection, and the protection afforded by 2·5 per cent dextrose containing 0·05 per cent B.A.L. was almost certainly the effect of the sugar.

### DISCUSSION.

The object of this work was to find suitable conditions for the preservation of fully active Rous virus on a large scale, the requirements being that the resuspended product should be non-aggregated and capable of being further purified by fractional centrifugation. The method now adopted, that of freeze-drying virus concentrates in Lemco broth, fulfils the condition, but little light has been thrown on the mode of action of the stabilizing material. Virus concentrates dried in this way have retained their full activity for minimum periods of 12 months.

The experiments with water alone showed that Dmochowski's (1948) results are confirmed in so far as virus may be recovered after drying—and our preparations had all been treated with 1:10,000 HCN during the isolation procedure—but it was not possible under these conditions to recover as much as 60 to 80 per cent of the initial virus activity as he claimed.

Salt solutions had no preservative action, and broth-containing media showed immediate advantages, even where the suspensions had been "purified" by centrifugal fractionation before desiccation. The action of the broth appears to be confined to the freezing-and-drying cycle or subsequently, since nothing was gained by carrying out the concentration stages in broth or broth-containing media, for which buffer-trypsin is quite satisfactory. It is unlikely, therefore, that the effect is solely an example of the phenomenon described by Adams (1948). The conditions of freeze-drying, moreover, do not involve vigorous shaking of the material but, equally, there is as yet no knowledge of the surface conditions which exist at an ice surface which is gradually receding through a drying mass. The observation that weaker (10 per cent) Lemco broth is a less effective protective agent is of interest in this respect.

It is possible to suppose that the action of broth and similar material is complex, that one or a combination of such factors as the following are important: (*a*) The broth provides optimum conditions during freezing—such as an optimum eutectic point; (*b*) that protection is conferred against the deleterious effect of heavy metals or of oxidation systems; (*c*) that, in some way, the tendency of the virus to aggregate is reduced; or (*d*) that an optimum amount of water only is lost. The interesting suggestion has been made that the preservative action of glycerol and sugar solutions in general is a result of water retention in or around the virus or bacterium (Kaiser, 1942). There appear to be no data available for the degree of dryness which has been achieved with other dried viruses. It may well be that it is possible to remove *irreversibly* water which is part of the internal structure of the body, with resultant inactivation.

Three, (*a*), (*b*) and (*d*), of the above four suggestions are rendered less probable by the fact that it appears to be possible to " reactivate " virus dried under unfavourable conditions by resuspending the dried material in Lemco broth.

The mode of action of broth and similar media is being further investigated.

### SUMMARY.

The preservation of Rous virus activity has been investigated after freezing-and-drying virus concentrates in a variety of media. In general, less highly purified suspensions are more readily preserved, and the tumour protein impurities apparently have a protective action. Broth-containing media have given optimum results, and full activity of the dried virus may be recovered after storage for periods of a year or more.

The authors wish to express their thanks to Professor A. Haddow for his interest and encouragement, and to Messrs. J. F. Thomas, J. Marsh and C. Smith for their technical assistance.

The investigation has been aided by the award of the Laura de Saliceto Studentship (1946–1950) to one of us (R. J. C. H.), and has been supported by grants to the Royal Cancer Hospital from the British Empire Cancer Campaign, the Anna Fuller Fund, the Jane Coffin Childs Memorial Fund for Medical Research, and the Division of Research Grants of the United States Public Health Service.

### REFERENCES.

ADAMS, M. H.—(1948) *J. gen. Physiol.*, **31**, 417.
BAUER, J. H., AND PICKELS, E. G.—(1940) *J. exp. Med.*, **71**, 83.
BEHRENS, C. A., AND FERGUSON, W. W.—(1935) *J. infect. Dis.*, **56**, 84.
CARR, J. G., AND HARRIS, R. J. C.—(1951) *Brit. J. Cancer*, **5**, 83.
CLAUDE, A.—(1937*a*) *J. exp. Med.*, **66**, 59.—(1937*b*) *Amer. J. Cancer*, **30**, 742.
*Idem* AND ROTHEN, A.—(1940) *J. exp. Med.*, **71**, 619.
DMOCHOWSKI, L.—(1948) *J. nat. Cancer Inst.*, **9**, 73.
HOFFSTADT, R. E., AND TRIPI, H. B.—(1946) *J. infect. Dis.*, **78**, 183.
JOHNSON, C. D., AND GOODPASTURE, E. W.—(1934) *J. exp. Med.*, **59**, 1.
KAISER, M.—(1942) *Biol. Gen.* (*Vienna*), **16**, 513.
KITCHEN, S. F.—(1934) *Amer. J. trop. Med.*, **14**, 457.
KNOX, R.—(1939) *J. Path. Bact.*, **49**, 467.
MCCULLAGH, D. R., CASSIDY, J. W., VALENTINE, F., AND TOLKSDORF, S.—(1949) *Proc. Soc. exp. Biol., N.Y.*, **71**, 295.
MCILVAINE, T. C.—(1921) *J. biol. Chem.*, **49**, 183.
RIVERS, T. M., AND WARD, S. M.—(1935) *J. exp. Med.*, **62**, 549.
ROUS, P.—(1911) *Ibid.*, **13**, 397.
*Idem* AND MURPHY, J. B.—(1914) *Ibid.*, **19**, 52.
SAWYER, W. A., LLOYD, W. D. M., AND KITCHEN, S. F.—(1929) *Ibid.*, **50**, 1.
SCHADE, A. L.—(1945) ' Amer. Ass. Adv. Sci. Res. Conference on Cancer,' p. 58.
SCHERP, H. W., FLOSDORF, E. W., AND SHAW, D. R.—(1933), *J. Immunol.*, **34**, 447.
SHOPE, R. E.—(1931), *J. exp. Med.*, **54**, 373.